# Flies

**Published:** 1914-09

**Authors:** 


**A Monthly Review of Medicine and Surgery**

EDITOR AND PUBLISHER, DR. A. L. BENEDICT, 228 Summer St., corner of Elmwood Ave., Buffalo, (Address for all communications. Please make personal and telephone calls before 1 P. M.)

Third, in age, on the western continent. Independent, but supports professional organization. News limited mainly to Western N. Y. and Penn.

Subscription $2.00 a year; $3.00 for two years in advance. Changes and errors in address or failure or duplication of delivery, should be reported immediately.

Yearly Volume 70. SEPTEMBER, 1914. No. 2

### Flies.

Flies are, admittedly, a danger on account of their tendency to transmit infectious matter from various kinds of filth. This being the case, we object strenuously to enlisting the services of children in the fly campaign. Children are, generally speaking, the least resistant to infections, the least able to observe sanitary precautions, the least aesthetic, of the entire population. Still more do we object to the recent movement to encourage children to take risks as fly-killers, by making dead flies currency, for admission to picture shows, etc., or for bounties. We do not like to think of any child hoarding and swapping flies like picture cards, postage stamps and cigarette coupons.

